# Disseminated *Mycobacterium abscessus* subsp. *massiliense* infection in a Good’s syndrome patient negative for human immunodeficiency virus and anti-interferon-γ autoantibody: a case report

**DOI:** 10.1186/s12879-020-05136-x

**Published:** 2020-06-20

**Authors:** Waki Imoto, Koichi Yamada, Yuriko Hajika, Kousuke Okamoto, Yuka Myodo, Makoto Niki, Gaku Kuwabara, Kazushi Yamairi, Wataru Shibata, Naoko Yoshii, Kiyotaka Nakaie, Kazutaka Yoshizawa, Hiroki Namikawa, Tetsuya Watanabe, Kazuhisa Asai, Hiroshi Moro, Yukihiro Kaneko, Tomoya Kawaguchi, Yoshiaki Itoh, Hiroshi Kakeya

**Affiliations:** 1 Department of Infection Control Science, Osaka City University Graduate School of Medicine, 1-4-3, Asahi-machi, Abeno-ku, Osaka, 545-8585 Japan; 2 Department of Infectious Disease Medicine, Osaka City University Hospital, 1-5-7 Asahi-machi, Abeno-ku, Osaka, 545-8586 Japan; 3 Department of Respiratory Medicine, Osaka City University Graduate School of Medicine, 1-4-3 Asahi-machi, Abeno-ku, Osaka, 545-8585 Japan; 4 Department of Infection Control and Prevention, Osaka City University Hospital, 1-5-7 Asahi-machi, Abeno-ku, Osaka, 545-8586 Japan; 5 Department of Metabolism, Endocrinology, Osaka City University Graduate School of Medicine, 1-4-3 Asahi-machi, Abeno-ku, Osaka, 545-8585 Japan; 6 Department of Neurology, Osaka City University Graduate School of Medicine, 1-4-3 Asahi-machi, Abeno-ku, Osaka, 545-8585 Japan; 7 Department of Respiratory Medicine and Infectious Diseases, Niigata University Graduate School of Medical and Dental Sciences, 1-757 Asahimachi-dori, Chuo-ku, Niigata, 951-8510 Japan; 8 Department of Medical Education and General Practice, Osaka City University Graduate School of Medicine, 1-4-3 Asahi-machi, Abeno-ku, Osaka, 545-8585 Japan; 9 Department of Bacteriology, Osaka City University Graduate School of Medicine, 1-4-3 Asahi-machi, Abeno-ku, Osaka, 545-8585 Japan

**Keywords:** Anti-interferon-gamma autoantibody, Disseminated, Good’s syndrome, *Mycobacterium abscessus*, Nontuberculous mycobacterial infection

## Abstract

**Background:**

Good’s syndrome (GS) is characterized by immunodeficiency, and can lead to severe infection, which is the most significant complication. Although *Mycobacterium* rarely causes infection in patients with GS, disseminated nontuberculous mycobacterial (NTM) infection frequently occurs in GS patients that are also positive for the human immunodeficiency virus (HIV) or anti-interferon (IFN)-γ autoantibodies. Here, we report a rare case of GS with NTM without HIV or IFN-γ autoantibodies.

**Case presentation:**

A 57-year-old Japanese male with GS and myasthenia gravis (treated with prednisolone and tacrolimus) was diagnosed with disseminated NTM infection caused by *Mycobacterium abscessus* subsp. *massiliense*. He presented with fever and back pain. Blood, lumbar tissue, urine, stool, and sputum cultures tested positive for *M. abscessus*. Bacteremia, spondylitis, intestinal lumber abscess, and lung infection were confirmed by bacteriological examination and diagnostic imaging; urinary and intestinal tract infections were suspected by bacteriological examination but not confirmed by imaging. Despite multidrug combination therapy, including azithromycin, imipenem/cilastatin, levofloxacin, minocycline, linezolid, and sitafloxacin, the patient ultimately died of the infection. The patient tested negative for HIV and anti-IFN-γ autoantibodies.

**Conclusions:**

Since myasthenia gravis symptoms interfere with therapy, patients with GS and their physicians should carefully consider the antibacterial treatment options against disseminated NTM.

## Background

Disseminated nontuberculous mycobacterial (NTM) infection is often associated with an infection by the human immunodeficiency virus (HIV) [1]. Anti-interferon-gamma (IFN-γ) autoantibodies are typically found in patients with disseminated NTM infection without HIV infection, especially in the Asian population [1, 2]. Good’s syndrome (GS) is characterized by thymoma, hypogammaglobulinemia, and multiple infections [3]. Despite a few cases of *Mycobacterium* infection associated with thymoma, to the best of our knowledge, there has been no report of *Mycobacterium* associated with GS [4–6]. Herein, we describe a rare case of GS with disseminated NTM infection.

## Case presentation

The patient was a 57-year-old Japanese male diagnosed with GS (thymoma and hypogammaglobulinemia), myasthenia gravis with anti-striational antibodies, and type 2 diabetes. Prednisolone (PSL) and tacrolimus (TAC) were used to treat the myasthenia gravis for more than 5 years, and his thymoma was removed at the age of 27 years. His father had been treated for lung *Mycobacterium tuberculosis* infection. The patient smoked for 27 years (since his twenties) and was a social drinker. He had worked in specimen processing at a specimen inspection company. His daily routine involved spending most of the day in bed and required assistance with his wheelchair and meals.

**Fig. 1:**
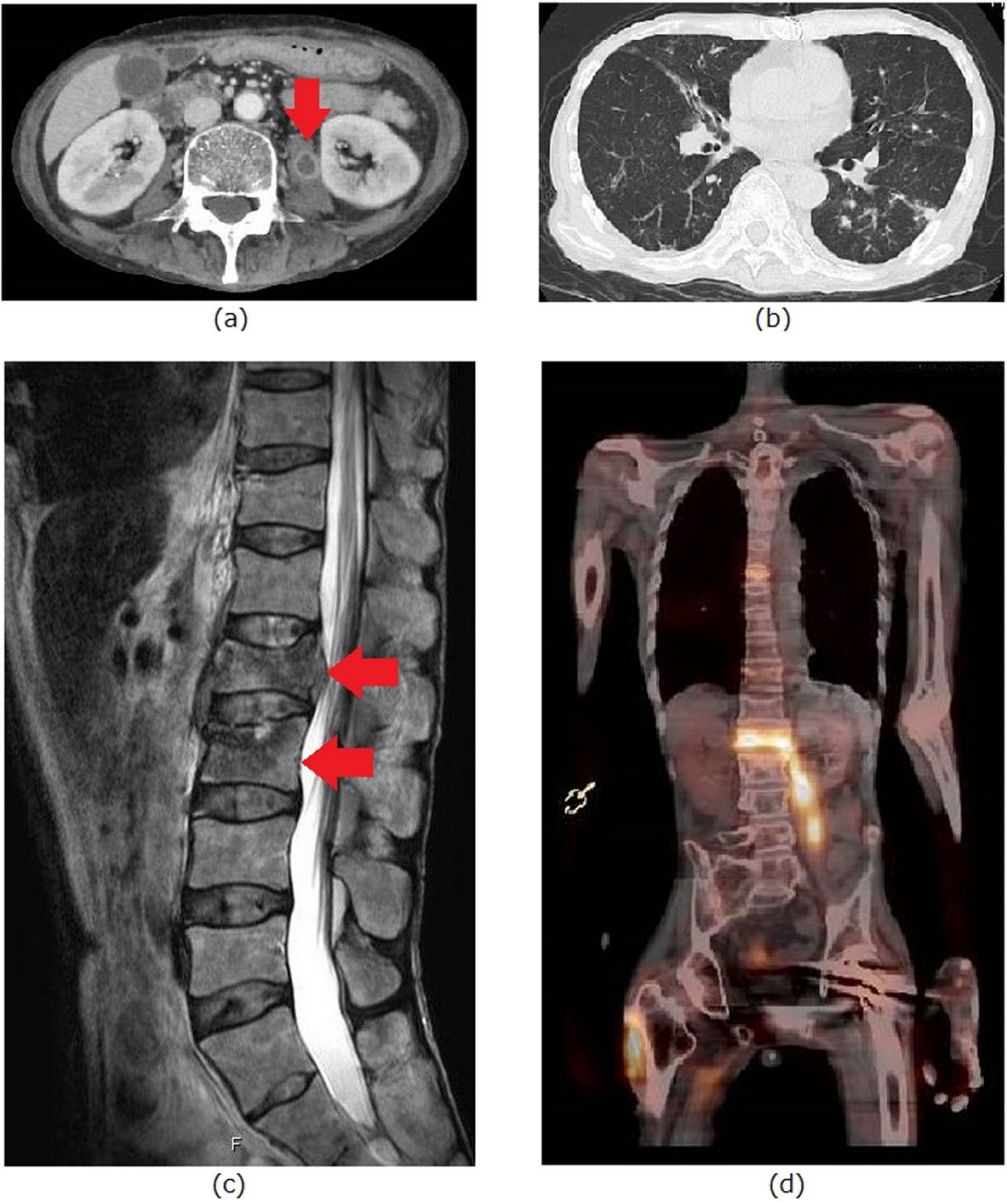
**a** Contrast-enhanced computed tomography scan of the abdomen showing the left iliopsoas abscess (red arrow). **b** Chest computed tomography showing the scattered nodules. **c** Contrast-enhanced magnetic resonance imaging of the spine (T2) showing pyogenic spondylitis at lumbar segments 1 and 2 (red arrows). **d** Gallium scintigraphy showing the accumulation of salt in the lumbar spine and iliopsoas muscle

The patient presented with fever and back pain 1 month before his outpatient visit at the Department of Neurology. He was hospitalized during his regular visit, at which point blood and sputum samples were collected for culture, and he was administered tazobactam/piperacillin (TAZ/PIPC) and immunoglobulin by his primary care physician. The sputum smear was positive for acid-fast bacilli; chest computed tomography showed a suspected lung NTM infection and lumbar intestinal abscess, and magnetic resonance imaging revealed spondylitis (lumbar segments 1–2) during hospitalization (Fig. 1). Infective endocarditis was not detected by transthoracic echocardiography. Mycobacterial infection was suspected, and blood culture was performed on day 5 of hospitalization. His general condition and vitals were stable, and the TAZ/PIPC treatment was continued while awaiting empirical therapy for *Mycobacteria*. Two days later, blood and sputum cultures revealed the presence of *Mycobacterium abscessus,* resulting in a diagnosis of disseminated NTM infection.

The patient was transferred to the Department of Infectious Disease and was screened for immunodeficiency. He tested negative for HIV-specific antibodies, and the blood samples sent to Nigata University tested negative for anti-IFN-γ autoantibodies. The conclusive diagnosis of *M. abscessus* subsp. *massiliense* infection was the result of a combination of 16S ribosomal RNA sequencing and nucleic acid chromatography of the RNA polymerase *B* and *hsp65* genes. He underwent a lumbar biopsy on day 8 after hospitalization and was prescribed empiric therapy with imipenem (IPM)/cilastatin, levofloxacin, and azithromycin. The antibiotics were continued since *M. abscessus* was also detected in the biopsy tissue, urine, and stool cultures.

**Fig. 2:**
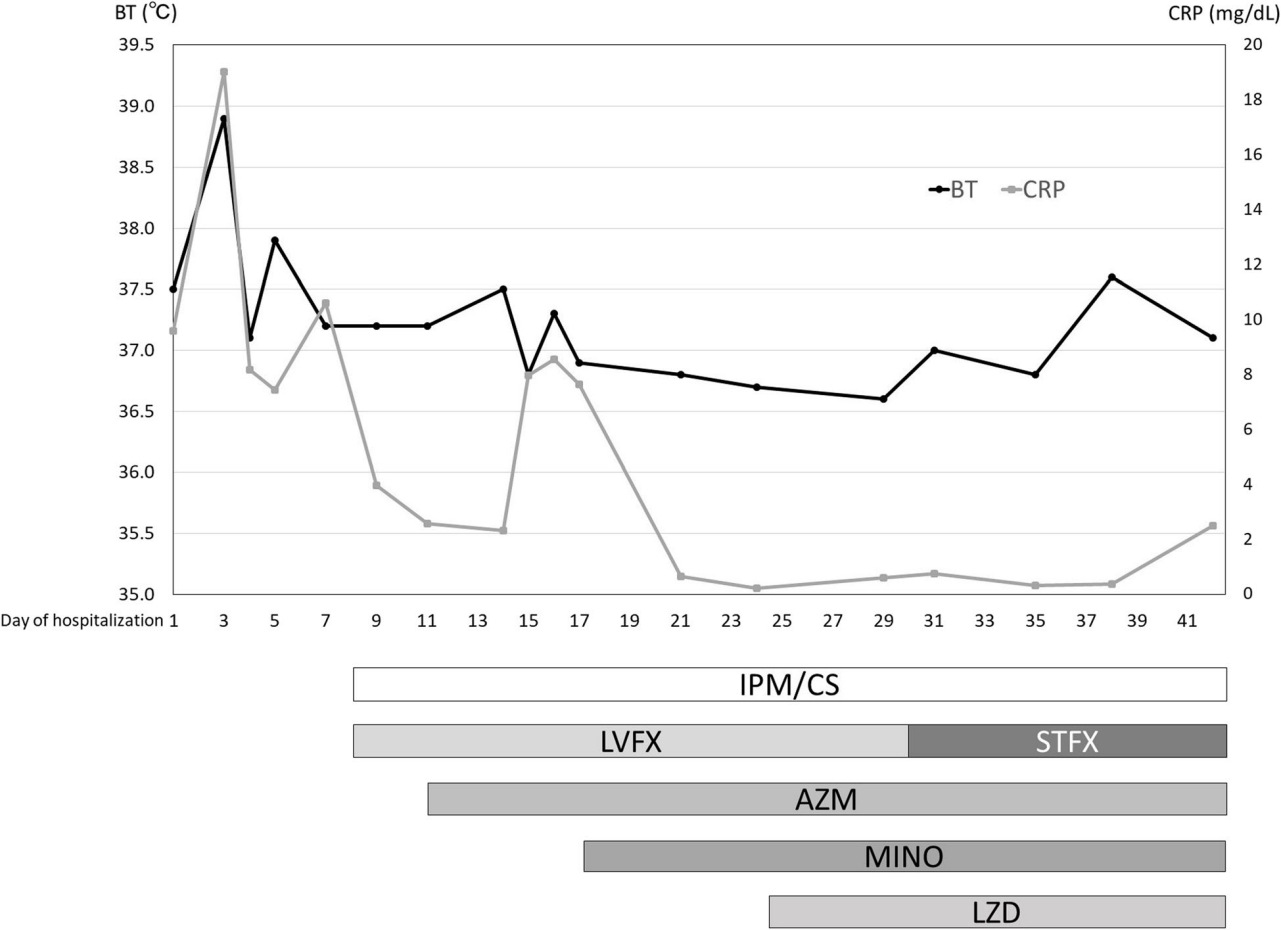
The clinical course of the present case. Transition of body temperature (black line) and C-reactive protein (grey line) relative to the antibiotic treatment regimen. AZM, azithromycin; BT, body temperature; CRP, C-reactive protein; IPM/CS, imipenem/cilastatin; LVFX, levofloxacin; LZD, linezolid; STMINO, minocycline; FX, sitafloxacin

The patient developed a gastrointestinal (rectum and descending colon) perforation on day 15 of hospitalization and underwent surgery (high anterior resection, left hemicolectomy, colostomy, and abdominal drainage). Despite the continued use of antibiotics, his spondylitis worsened. Minocycline (MINO) and linezolid (LZD) were included in the antibiotic regimen on day 17 and 24, respectively. Finally, after levofloxacin was substituted with sitafloxacin (STFX), the antibiotic combination of IPM/CS, STFX, azithromycin, MINO, and LZD was continued (Fig. 2). Although the sensitivity of *M. abscessus* subsp. *massiliense* was detected by microdilution [7, 8], the strain showed a different susceptibility towards each antibiotic (Table 1), and the patient’s general condition worsened. The patient and his family were informed of the treatment options, and after obtaining consent, he was moved to palliative care. He passed away on day 49 in the hospital.

**Table 1:** Antibiotic susceptibility of the identified *Mycobacterium abscessus* subsp. *massiliense* clinical isolate

Antibiotic	Minimum Inhibitory Concentration Breakpoints (mg/L) [7]			Minimum Inhibitory Concentration (mg/L)
	Susceptibility	Intermediate	Resistance	
Clarithromycin	≦2	4	≧8	8
Imipenem	≦4	8–16	≧32	16
Meropenem	≦4	8–16	≧32	> 64
Minocycline	≦1	2–4	≧8	> 8
Amikacin	≦16	32	≧64	16
Moxifloxacin	≦1	2	≧4	> 8
Linezolid	≦8	16	≧32	32
Trimethoprim-sulfamethoxazole	≦2/38	–	≧4/76	> 8/152

## Discussion and conclusions

Infections are the most frequent and important complications associated with GS [9]. Previous reports have shown that the upper and lower respiratory tracts are the most common sites of infection in patients with GS [10], and bacterial infections are the most significant [9, 10]. However, infections by *Mycobacterium* are rare in GS patients, and only a few cases of *Mycobacterium* infection associated with thymoma (without GS) have been reported [4–6, 11]. We surveyed the literature, and the relevant reports are listed in Table 2. To the best of our knowledge, this is the first report of a *Mycobacterium* infection associated with GS.

**Table 2:** Past reports of thymoma (with or without Good’s syndrome) with *Mycobacterium* infection

	This case	Murakami O, et al. [4]	Satoh H, et al. [5]
GS or thymoma	GS	thymoma	thymoma (GS suspected, not detected)
age	57 y	33 y	69 y
sex	Male	Male	Male
Infectious microorganism	*Mycobacterium abscessus* subsp. *massiliense*	*Mycobacterium tuberculosis* suspected	*Mycobacterium intracellulare*
treatment for infection	IPM, AZM, MINO, LZD, STFX	INH, RFP, SM	observation
complications	DM, anti-rhabdomyolysis, antibody-positive myasthenia gravis	not particular	not particular
immunosuppressants	PSL, TAC	none	none
year	2019	1998	2012

*AZM* azithromycin, *DM* diabetes mellitus, *GS* Good’s syndrome, *INH* Isoniazid, *IPM* imipenem, *LZD* linezolid, *MINO* minocycline, *PSL* prednisolone, *RFP* Rifampicin, *SM* Streptomycin

TAC: tacrolimus

A common variable immune deficiency like GS is characterized by hypogammaglobulinemia, which increases the risk of infection by bacteria and certain viruses [12]. In particular, infection by cytomegaloviruses and *Pneumocystis jirovecii* is rampant in GS patients since their immune cells are depleted; this is a common characteristic of GS that distinguishes it from common variable immune deficiency [13, 14]. Despite the depletion of immune cells, as the major host defense mechanism, mycobacterial infections are nevertheless rare in GS patients [15]; however, the reason remains to be elucidated [11]. The severe disseminated NTM infection in our GS patient might have been induced by the several treatments he was administered. The patient had myasthenia gravis, which was treated with PSL and TAC. These immunosuppressive agents are known to induce cell-mediated immunodeficiency, which might have triggered the mycobacterial infection [16].

Treatment was difficult in the present case because of the immunodeficiency and side effects of the drugs. Liu and Hu [17] reported that aminoglycosides might worsen myasthenia gravis by competitively inhibiting the release of acetylcholine from the presynaptic membranes, thereby impairing depolarization of the postsynaptic membrane and reducing irritability of the myocyte membrane around the end-plate membrane that subsequently leads to blockade of the neuromuscular junction. Thus, even though aminoglycosides are very potent antimicrobial agents, they could not be used to treat our patient [18]. Moreover, in the past, *M. abscessus* was reported to show the lowest macrolide resistance rate [19]. However, in this case, the strain showed a high MIC (MIC = 8) value for clarithromycin, which remarkably challenged treatment. There is no clear association between the intestine perforation and *Mycobacterium* infection of the present case. The resected specimen did not show any abnormalities; thus, *Mycobacterium* or other infections could not be diagnosed. Since *M. abscessus* was detected in the stool culture, it is still possible that *M. abscessus* caused the intestinal perforation.

In conclusion, this is the first case of disseminated NTM infection in a GS patient without infection by HIV or the production of anti-IFN-γ autoantibodies. Although NTM infection can occur in GS patients, the treatment for *M. abscessus* infection is difficult in such cases and should be carefully considered, since aminoglycosides cannot be used in GS.

## Data Availability

The data that support the findings of this study are available from the corresponding author upon reasonable request.

## Abbreviations

GS: Good’s syndrome
HIV: Human immunodeficiency virus
IFN-γ: Interferon-gamma
INH: Isoniazid
IPM: Imipenem
LZD: Linezolid
MINO: Minocycline
NTM: Nontuberculous mycobacterial
PSL: Prednisolone
RFP: Rifampicin
SM: Streptomycin
TAC: Tacrolimus
TAZ/PIPC: Tazobactam/piperacillin
